# Comment faisait-on jusqu'aux années 1980 avant l'ordinateur personnel ? Des trous, des petits trous

**DOI:** 10.48327/mtsi.v2i3.2022.253

**Published:** 2022-07-12

**Authors:** Jean-Loup REY

**Affiliations:** Le Barry, 04180 Villeneuve, France

Les ordinateurs pour les chercheurs de terrain ne sont apparus que dans les années 1980. Auparavant, on se servait des fiches perforées que les plus anciens connaissent; mais les champions de l'ordi, les moins de 60 ans, connaissent-ils les moyens artisanaux d'autrefois? Nous ne parlerons que des fiches (pré)perforées qui ont apporté un progrès net par rapport aux registres de tableaux et de colonnes. Ces fiches, dont il existait plusieurs tailles, étaient vendues sans impression mais il était possible d'en faire imprimer pour un objectif spécifique (Fig. 1, 2, 3, 4, 5).

**Figure 1:**
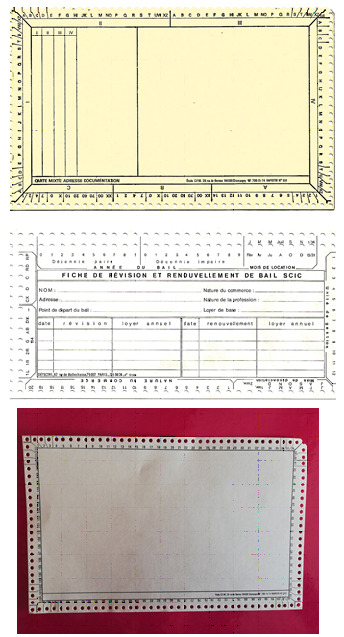
Exemples de fiches standards Examples of standard sheets

**Figure 2:**
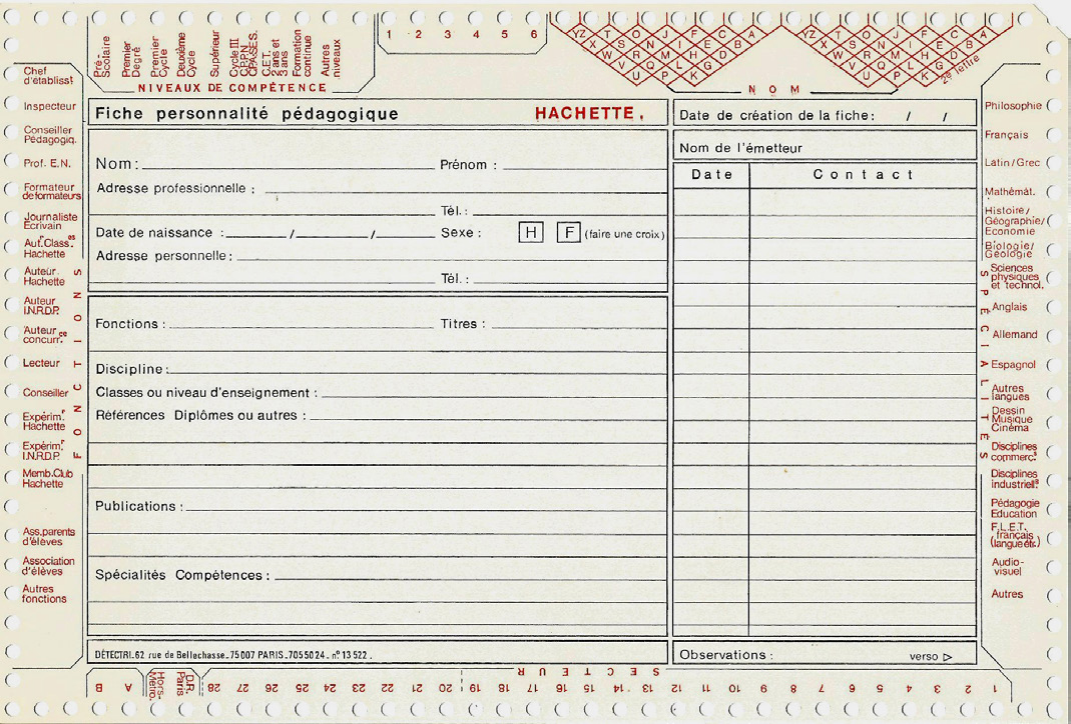
Exemple de fiche bibliothèque Example of a library sheet

**Figure 3:**
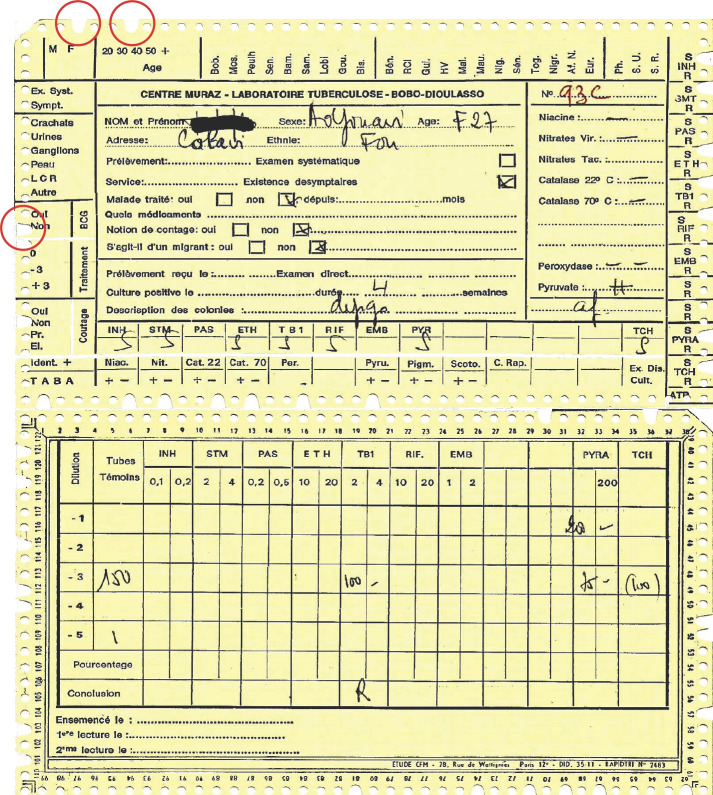
Recto verso des fiches utilisées au Centre Muraz à Bobo Dioulasso. 1^er^ cercle: encoche correspondant au sexe du patient, 2^e^ cercle: encoche correspondant à l’âge du patient (27), 3^e^ cercle: encoche pas de vaccination BCG Front and back of the sheets used at the Muraz Center in Bobo Dioulasso. 1^st^ circle: notch corresponding to the patient's sex, 2^nd^ circle: notch corresponding to the patient's age (27), 3^rd^ circle: notch no BCG vaccination

**Figure 4:**
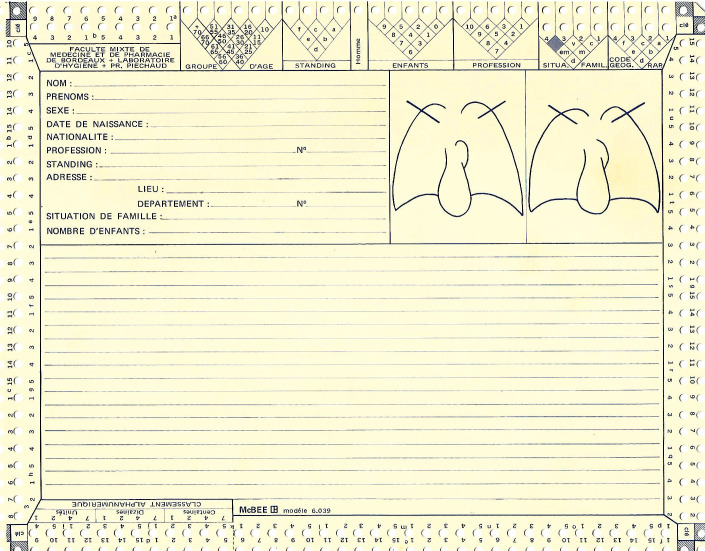
Exemple de fiche pour la tuberculose Example of sheet for tuberculosis

**Figure 5:**
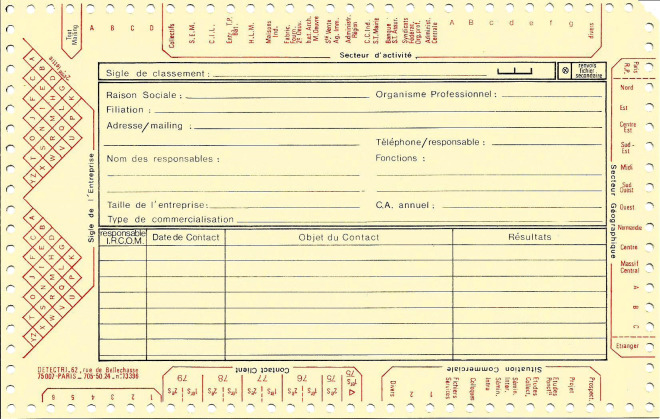
Exemple de fiche client Example of a customer sheet

Leur utilisation nécessitait une pince coupante pour ouvrir la perforation sur le bord de la fiche. Il existait pour cela des pinces spéciales. Mais le PMU (Pari mutuel urbain) utilisant le même système, il était facile de se procurer gratuitement ces petites pinces adéquates (Fig. 6). Les entailles correspondaient au signe positif choisi et recueilli lors de l'enquête de terrain ou l’étude au laboratoire. Quand toutes les fiches étaient prêtes, on les rassemblait par effectif d'une cinquantaine, on passait une aiguille à tricoter dans le trou souhaité, puis on agitait. Toutes les fiches qui tombaient possédaient le signe recherché (Fig. 7). Il était possible de renouveler l'opération pour obtenir des sous-groupes porteurs de 2 signes, ou plus.

**Figure 6:**
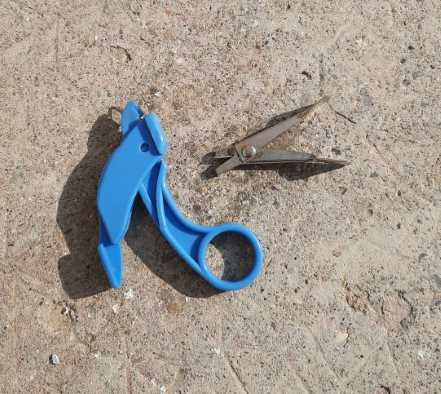
À droite pince PMU, à gauche pince commerciale On the right PMU punch, on the left commercial punch

**Figure 7:**
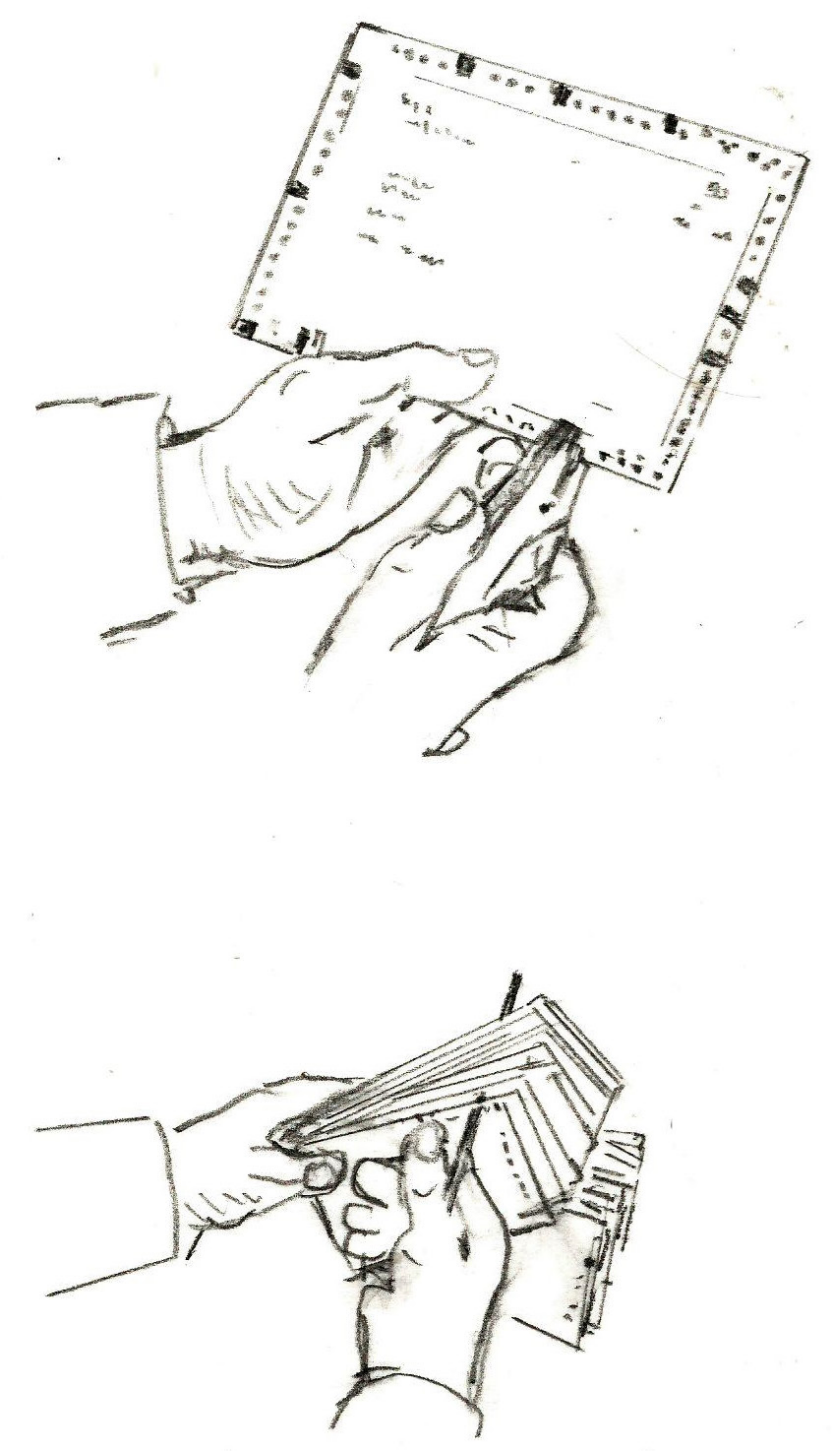
Dessins des procédures d'encochage et de tri des fiches Drawings of notching and sorting procedures

Dans les archives il existe encore des cartons de fiches sur les trypanosomés du secteur Lobi au Burkina Faso, sur les souches de mycobactéries ou de méningocoques du Centre Muraz, et bien d'autres (Fig. 2, 3).

Ces fiches existaient depuis un certain temps. En 1725, un certain Basile Bouchon avait inventé le concept de la carte perforée, qui fut d'abord employée pour les orgues de barbarie. Dès 1728, des cartes perforées reliées entre elles furent utilisées dans les métiers à tisser Jacquard [1, 2].

Plus tard, le mathématicien Charles Babbage eut l'idée de recourir aux cartes perforées du métier à tisser pour une machine destinée à calculer l'impression de tables mathématiques. Le prototype de cette machine analytique fut repris par son fils qui en fit la démonstration à l'Académie royale d'astronomie de Londres en 1908 [3]. Cette machine a été considérée comme l'ancêtre de l'ordinateur.

## Liens D'intérêts

L'auteur ne déclare aucun lien d'intérêt.
